# Interstitial HDR brachytherapy for anal cancer—results and quality of life

**DOI:** 10.1007/s00066-024-02316-5

**Published:** 2024-11-14

**Authors:** Michaela Jirkovská, Hana Stankušová, Anna Kindlová, Daniel Jirkovský, Radka Lohynská

**Affiliations:** 1 Department of Oncology, Second Faculty of Medicine, Charles University and Motol University Hospital, V Úvalu 84, 150 06 Prague, Czech Republic; 2 Department of Oncology, First Faculty of Medicine, Charles University and Thomayer University Hospital in Prague, Vídeňská 800, 140 59 Prague, Czech Republic; 3 Second Faculty of Medicine, Charles University in Prague, V Úvalu 84, 150 06 Prague, Czech Republic

**Keywords:** Squamous cell carcinoma of the anus, Radiochemotherapy, Interstitial brachytherapy, Long-term outcomes, Late toxicity

## Abstract

**Purpose:**

While anal cancer is a very rare oncological diagnosis representing less than 2% of lower gastrointestinal tract cancers, the incidence has doubled in the past 20 years. Radical radiochemotherapy with sequential or simultaneous boost is now the standard treatment modality. Interstitial HDR brachytherapy is one of the boost application options. Implementation of new radiotherapy techniques has resulted in improved therapeutic outcomes; however, it is still associated with acute and especially late toxicity. Gastrointestinal disorders and sexual dysfunction are the most frequent factors affecting the long-term quality of cured patients’ lives.

**Methods:**

A total of 96 patients consecutively treated between 2000 and 2022 with external beam radio-/chemotherapy and an interstitial brachytherapy boost for histologically verified nonmetastatic anal squamous cell carcinoma were evaluated. The median follow-up time was 15.4 years (range 13.4–17.3 years). The primary objective of the study was to assess local control (LC) and quality of life (QoL). The Czech versions of internationally validated EORTC questionnaires were used to evaluate life quality—the basic EORTC QOL-C30 v.3 and the specific QOL-ANL 27 questionnaire.

**Results:**

Local control was 85.5% at 5 years, 83.4% at 10 years, 83.4% at 15 years, and 83.4% at 20 years, and there was no dependence on clinical stage. The most common forms of acute toxicity were cutaneous and hematological but were gastrointestinal for late toxicities. In the evaluation of quality of life, 80.5% of patients alive at the time participated. In the EORTC quality of life questionnaire C30 v.3, patients rated the functional scale score as 86.2 points (standard deviation [SD] = 12.6) and the symptom score as 15.5 points (SD = 12.5). The global health score achieved 68.4 points (SD = 23.6). The most common symptoms were fatigue with 25.6 points (SD = 20.2) and diarrhea with 19.0 points (SD = 27.8). In the QOL-ANL 27 questionnaire, symptom scales assessing bowel symptoms were scored 27.5 points (SD = 19) in non-stoma patients and 11.9 points (SD = 17.2) in stoma patients. In the single-item symptom scales, the highest scores were rated for frequency of urination with 26.4 points (SD = 30.8), need to be close to a toilet with 22.4 points (SD = 27.3), and self-cleaning more often with 25.3 points (SD = 31.8). In the functional scales assessing sex life and interest, men and women reported scores of 45.2 (SD = 23) and 45.5 points (SD = 19), respectively.

**Conclusion:**

Boost with interstitial HDR brachytherapy is an established safe method of anal cancer treatment, with excellent results and limited late toxicity. Functioning scales were rated relatively highly in QoL questionnaires, and the overall global health score was comparable to published data. Gastrointestinal difficulties, fatigue, and sexual dysfunction dominated the symptom scales in our cohort.

**Supplementary Information:**

The online version of this article (10.1007/s00066-024-02316-5) contains supplementary material, which is available to authorized users.

## Introduction

Anal cancer (AC) is a very rare oncological diagnosis. With an incidence of 0.7 to 1.9 new cases per 100,000 population per year, it is responsible for less than 2% of lower gastrointestinal tract cancers [1]. Women are affected more often than men [2, 3]. The incidence of AC has doubled in all demographic groups over the past 20 years [4]. The main causes are the increasing incidence and persistence of high-risk human papillomaviruses (HPV) [5], the increasing prevalence of the human immunodeficiency virus (HIV), and changes in sexual behavior [6]. AC is a predominantly locoregional disease (only 10% of patients have distant metastases at the time of diagnosis), with the overall relative survival improving slightly over the years [1]. Squamous cell carcinoma accounts for 80–85% of all cases [7], and the HPV status has important prognostic value [8, 9]. Among the staging procedures, whole-body CT with fluorodeoxyglucose positron-emission tomography (FDG-PET/CT) plays a key role as a prognostic factor [10, 11] and in target volume definition during radiotherapy planning [12].

Surgery in terms of abdominoperineal resection has historically been the only therapeutic approach. However, apart from significant morbidity, it has not achieved fully satisfactory therapeutic results in terms of local control [13, 14]. Following encouraging first experiences in the 1970s [15], radical radiochemotherapy was established as the primary treatment modality and later based on randomized phase III clinical trials [16–21]. The combination of external beam radiotherapy (EBRT) and chemotherapy has been shown to be superior to radiotherapy alone [16, 22], even in the older population, independent of the combined Charlson comorbidity score [22, 23]. Implementation the of new RT techniques of intensity-modulated radiotherapy (IMRT) and image-guided radiotherapy (IGRT) has further improved outcomes, particularly in terms of colostomy-free survival (CFS) and reduced toxicity [24, 25]. Although the most conducive dose of RT in AC treatment remains unclear, particularly the treatment of locally advanced tumors may benefit from local dose escalation [26, 27]. Boost can be applied by EBRT sequentially or simultaneously or by interstitial brachytherapy (ISBT).

In contrast to an EBRT boost, adaptive brachytherapy (BT), as a highly conformal RT method, allows targeted radiation of the residual tumor, thus reducing toxicity to the organs at risk (OAR) [28]. Boost with high dose rate (HDR) ISBT allows late toxicity to be reduced and the quality of life of long-term surviving patients to be improved without affecting the therapeutic outcome [29].

The aim of our study was to evaluate treatment outcomes, toxicity, and quality of life (QoL) of patients treated with radio-/chemotherapy and an ISBT boost. Our hypothesis was that QoL scores would be influenced by the extent of disease and the age of patients and would improve over time.

## Materials and methods

### Study design and participants

In a retrospective single-institution study, a total of 96 patients consecutively treated between 2000 and 2022 with external beam radio-/chemotherapy and an ISBT boost for histologically verified nonmetastatic anal cancer were evaluated. All patients signed informed consent to treatment.

### Examination

Pretreatment examinations comprised a complete medical history including assessment of smoking and alcohol abuse. Physical examination and a local anorectal examination in the gynecological position were performed at the start and at the end of external beam radiotherapy by a brachytherapist. The diagnostic workup included rectoscopy and colonoscopy with biopsy and histological verification. All patients were evaluated by an imaging modality: computed tomography (CT) in 41% of patients, pelvic magnetic resonance imaging (MRI) in 16%, and PET/CT in 43%. The extent of disease was rated using the current TNM classification of malignancies and restaged according to the 8th edition for the study purposes.

### Treatment protocol

**Fig. 1:**
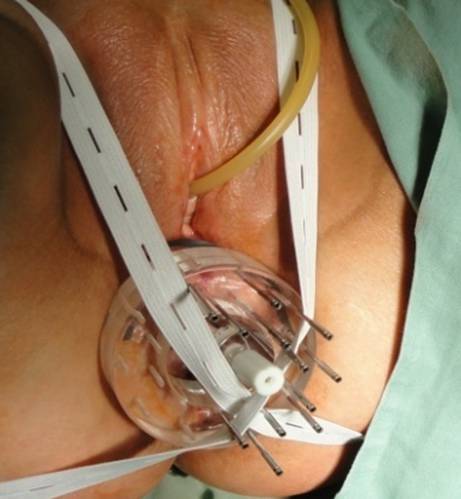
Implantation procedure: double-plane circular template with metal needles and anal cylinder

**Fig. 2:**
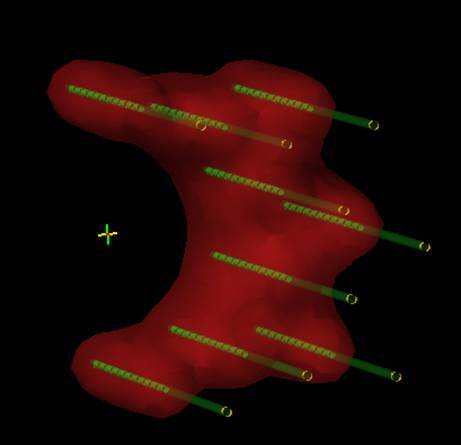
The Paris dosimetry system-double plane implant

**Fig. 3:**
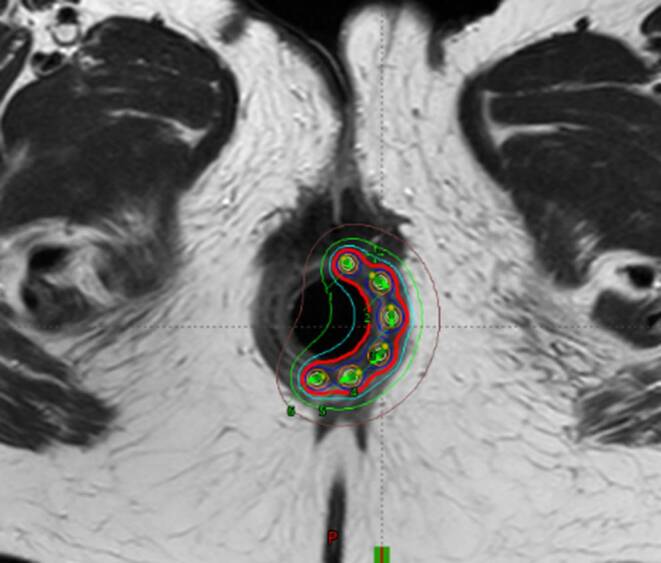
Dose distribution analysis on the post implant MRI scan

The planning CT and the irradiation were accomplished in the supine position. Contouring of target volumes and critical organs was performed in accordance with ICRU rules and published guidelines. Besides the usual OAR (bladder, external genitals, bowel bag, femoral heads), bone marrow was contoured in the irradiated volume. Patients were treated with EBRT up to a dose of 45 Gy in 25 fractions to the area of the primary tumor and the locoregional pelvic lymph nodes. All metastatic nodes were boosted with a sequential or simultaneous integrated boost up to a dose of 55 Gy. Suitable patients were indicated for concomitant chemotherapy. The most used regimen was mitomycin and 5‑flurouracil in a continuous infusion or capecitabine administered in the oral form. Subsequently, a boost using interstitial brachytherapy (ISBT) was applied under general anesthesia to the area of residual tumor. The condition was a tumor occupying no more than half of the anal canal circumference with a thickness ≤ 15 mm at the time of ISBT. Most commonly, 2 fractions of 5 Gy 1–2 times a week were indicated (76% of patients), less often 6–7 Gy in 2 fractions (24% of patients). ISBT application was performed using a single- or double-plane circular template and an anal cylinder with metal or plastic needles (Fig. 1) in a geometry according to the Paris system (Fig. 2). Orthogonal radiographs or MRI (Fig. 3) were used for planning. The CTV was defined by physical examination, initial endoscopy, and tumor extent on imaging. Only one patient (1%) received ISBT as a single modality, and 6 fractions of 7 Gy were prescribed.

### Follow-up and toxicity

Patients were followed up weekly during RT and assessed for acute toxicity according to CTCAE v.5. Subsequently, patients were followed up at 3‑month intervals for the first and second year, at 6‑month intervals until the fifth year, and at yearly intervals thereafter. At each follow-up visit, local findings and late toxicity were assessed and blood tests were performed. Follow-up imaging was indicated 3 months after completion of RT and afterwards when recurrence of the underlying disease was suspected.

### Quality of life

Czech versions of the internationally validated EORTC questionnaires were used. The EORTC Quality of Life Group granted permission to employ these questionnaires in the academic quality of life study.

EORTC QOL-C30 v.3 is a basic questionnaire assessing the quality of life of cancer patients. It consists of 30 questions. The questionnaire contains five functional scales covering physical, role, emotional, cognitive, and social functioning. It also includes three symptom scales to assess fatigue, pain, nausea, and vomiting. It consists of a scale assessing quality of life and global health score, and six separate questions assessing dyspnea, insomnia, appetite loss, constipation, diarrhea, and financial difficulties. Each of the multi-item scales includes a different set of unique questions. Likert scales of 1–4 (1 being not at all, 4 being very) and 1–7 (1 very poor, 7 excellent) are used for responses, respectively.

This questionnaire C30 was supplemented by the QOL-ANL 27 questionnaire, which is designed for anal cancer patients after chemoradiotherapy. It consists of 27 questions. Symptom scales (higher scores indicate higher symptom levels) include five multi-item scales assessing bowel symptoms, pain, stoma care, and vaginal symptoms. There are also seven single-item scales assessing urinary frequency, the necessity to clean oneself more often, the need to be close to a toilet, leg/ankle swelling, activity planning, pain of intercourse, and erection problems. Functional scales (higher scores indicate better function) include two multi-item scales, namely male and female sexual functioning.

Patients were first invited to complete the questionnaires by telephone and if they agreed to participate in the study, they were asked to complete both of the questionnaires. A total of 72 patients were alive at the time of study, of whom 58 participated in the questionnaire analysis (80.5%). Given the QOL-ANL 27 questionnaire was published only recently, we cannot make a reasonable comparison with the baseline quality of life data.

### Outcomes and statistical analysis

The primary objective of the study was to assess local control (LC) and quality of life (QoL). Our hypothesis was that QoL would be influenced by the extent of disease, the technique of external beam RT, and the age of patients, and would improve over time. For this purpose, we chose a level of 5 years after treatment completion. LC was defined as the time from the start of RT to local disease recurrence. Other evaluated parameters were overall survival (OS), defined as the time from initiation of RT to the time of last follow-up or patient death, disease-free survival (DFS), and distant metastasis-free survival (MFS), defined as the time from initiation of radiotherapy to disease recurrence or distant dissemination.

Data were analyzed using SPSS statistical software version 29 (IBM Corp., Armonk, NY, USA), and *p*-values lower than 0.05 were considered statistically significant. Univariate analyses of survival were carried out by the Kaplan–Meier method.

Evaluation of the quality of life questionnaires was performed according to the scoring manual. According to the formulas, a raw score was calculated and transferred to a linear scale from 0 to 100. The transformation is necessary to compare the scales and symptoms that contain different numbers of questions. Higher scores on functional, quality of life, and global health scales represent a better status. Higher scores on symptom scales and symptoms themselves indicate more pronounced symptomatology.

Mean values were specified with standard deviations (SD). Associations between the QoL scores and study variables were assessed by Student’s *t-*test and Mann–Whitney U test according to the nature of the variables. A two-sided *p*-value of < 0.05 was considered statistically significant.

## Results

The study included 96 patients treated between 2000 and 2020 with external beam radiotherapy and interstitial HDR brachytherapy for AC. Patient and tumor characteristics are summarized in Table 1. The median follow-up time of patients was 15.4 years (range 13.4–17.3 years).

**Table 1:** Patient, tumor, and treatment characteristics

Variable	Number of patients	QoL
*Total number of patients*	96 (100%)	58 (80.5%), 72 live
Men	12 (12.5%)	9 (15.5%)
Women	84 (87.5%)	49 (84.5%)
Age at the time of treatment (years)	30–81 (mean 64)	37–77 (mean 62)
Age at the time of completion the questionnaires (years)	–	52–92 (mean 73)
*T stage*		
T1	20 (21%)	13 (22%)
T2	55 (57%)	33 (57%)
T3	17 (18%)	12 (20%)
T4	4 (4%)	1 (1%)
*N stage*		
N0	63 (66%)	37 (64%)
N1	33 (34%)	21 (36%)
*Clinical stage (TNM 8)*		
I	19 (20%)	12 (21%)
II	44 (46%)	25 (43%)
III	33 (34%)	21 (36%)
*Histology*		
SCC	93 (97%)	57 (98%)
Other	3 (3%)	1 (2%)
Smoking	27 (28%)	21 (36%)
Alcohol	15 (16%)	13 (22%)
*Treatment*		
Concomitant RCT + ISBT	71 (74%)	42 (72%)
RT + ISBT	24 (25%)	16 (28%)
BT	1 (1%)	0 (0%)
*Technique of EBRT*		
3D CRT	43 (45%)	31 (53%)
IMRT	52 (55%)	27 (47%)
Overall treatment time, months	15–163 (mean 60)	15–163 (mean 61)
*BT technique*		
Interstitial	90 (94%)	57 (98%)
Intracavitary	6 (6%)	1 (2%)
*Chemotherapy regimen*		
MMC + 5-FU	66 (69%)	39 (67%)
5‑FU	5 (5%)	3 (5%)
Without chemotherapy	25 (26%)	16 (28%)

*QoL* quality of life, *SCC* squamous cell carcinoma, *RCT* radiochemotherapy, *ISBT* interstitial brachytherapy, *BT* brachytherapy, *EBRT* external beam radiotherapy, *3DCRT* three-dimensional conformal radiotherapy, *IMRT* intensity-modulated radiotherapy, *MMC* mitomycin, *5‑FU* 5-fluorouracil

**Fig. 4:**
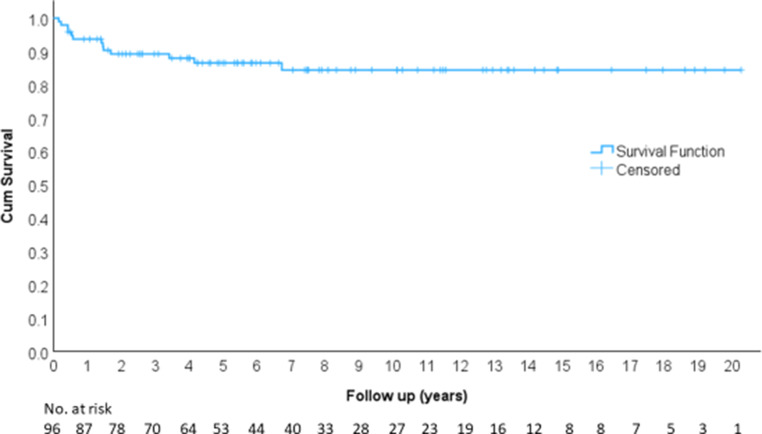
Local control

**Fig. 5:**
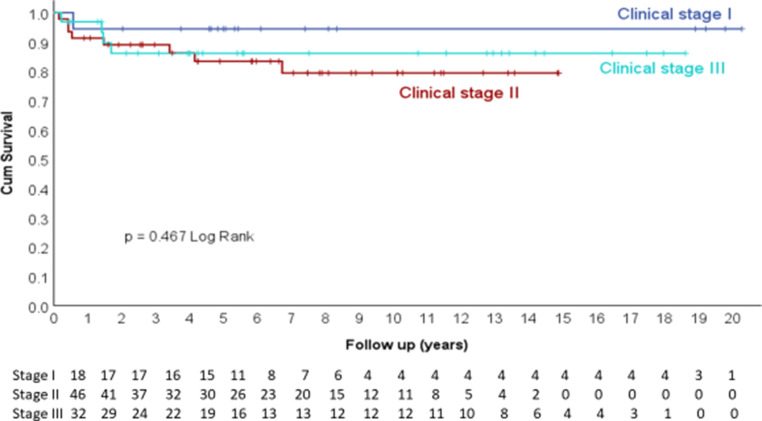
Local control according to clinical stage

Local control (LC) was 86.5% at 5 years, 84.4% at 10 years, 84.4% at 15 years, and 84.4% at 20 years (Fig. 4), and there was no dependence on clinical stage (Fig. 5).

**Fig. 6:**
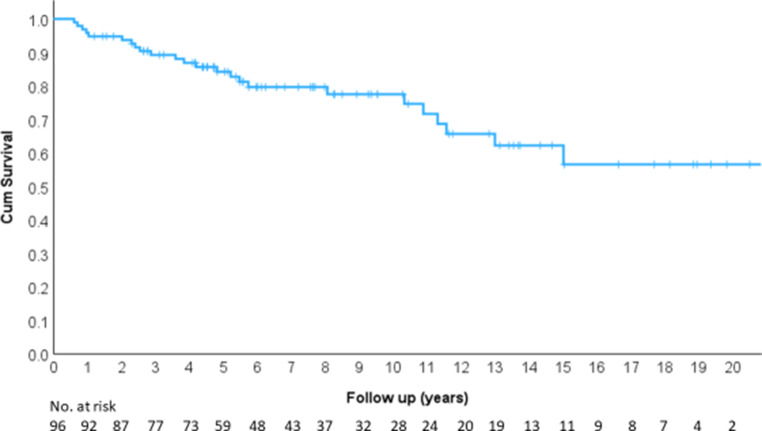
Overall survival

Overall survival (OS) was 84.2% at 5 years, 77.5% at 10 years, 62.2% at 15 years, and 56.6% at 20 years (Fig. 6).

**Fig. 7:**
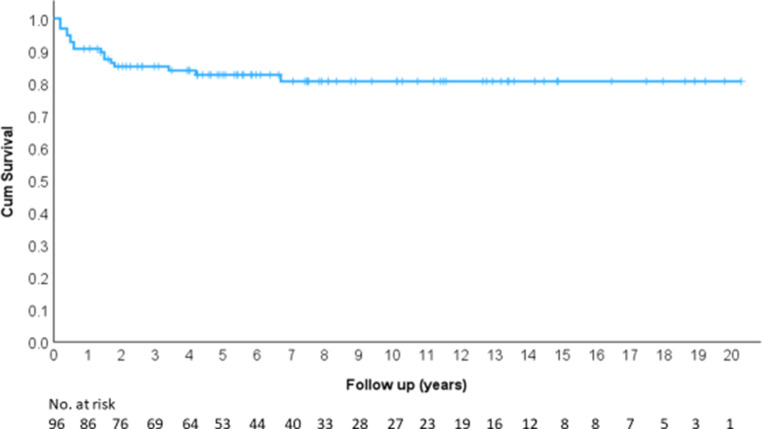
Disease-free survival

Disease-free survival (DFS) was 82.6% at 5 years, 80.6% at 10 years, 80.6% at 15 years, and 80.6% at 20 years (Fig. 7).

**Fig. 8:**
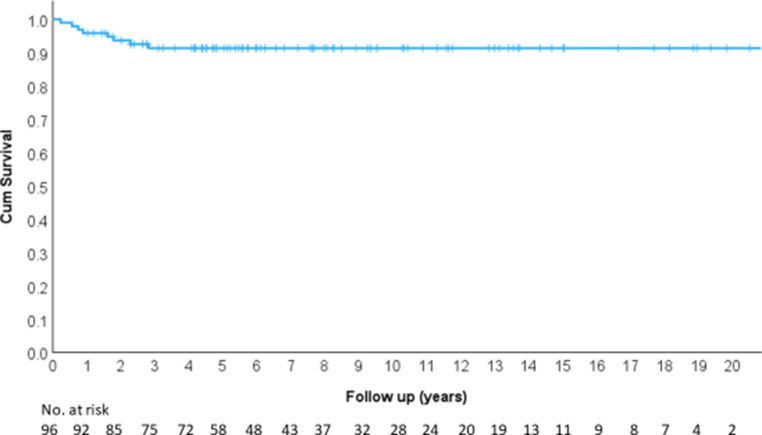
Metastasis-free survival

Distant metastasis-free survival (MFS) was 91.3% at 5 years, 91.3% at 10 years, 91.3% at 15 years, and 91.3% at 20 years (Fig. 8).

Colostomy-free survival (CFS) was 86.6% at 5 years, 83.6% at 10 years, 83.6% at 15 years, and 83.6% at 20 years.

**Fig. 9:**
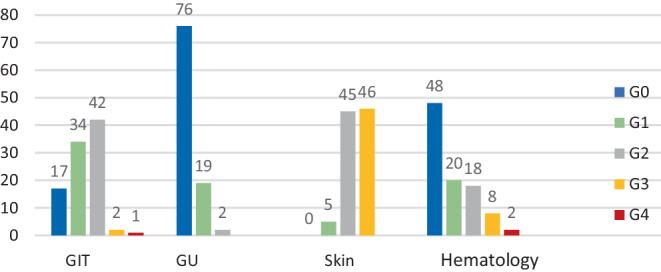
Acute toxicity. *GIT *gastrointestinal, *GU *genitourinary

Acute toxicity (Fig. 9) was evaluated according to the CTCAE criteria v.5 (transformed for the study). Gastrointestinal (GIT) toxicity grade (G)3–4 was reported by 3 patients (3%), but none of the evaluated patients developed genitourinary toxicity (GU) higher than G0–2. Skin toxicity, as the most common form of acute toxicity, occurred as G3 in 46 patients (48%). Hematological toxicity G3–4 was seen in 10 patients (10%).

**Fig. 10:**
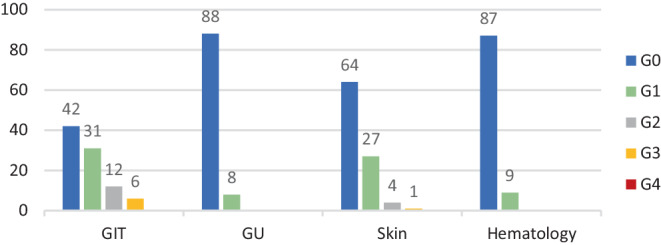
Late toxicity.* GIT *gastrointestinal, *GU *genitourinary

Late toxicity (Fig. 10) was assessed according to the CTCAE criteria v.5 (transformed for the study). Most of the patients had G0–2 toxicity, with only minimal higher grading. GIT toxicity G3 occurred in 6 patients, and GU toxicity was noted as G0–1 in all patients. Late skin toxicity was graded G3 in only one patient. Permanent stoma due to recurrence (10 patients) or a non-functioning sphincter (5 patients) was present in 15 patients (15.6%).

Quality of life was assessed by a single survey for at least 1 year after the end of treatment (range 1–20 years) and 80.5% of patients alive at that time participated. In the core questionnaire EORTC QOL-C30 v.3 (Supplementary materials—Table 2), patients rated on functional scales—overall functional score (FS) 86.2 (standard deviation [SD] = 12.6), physical functioning (PF2) 84.8 (SD = 16.8), role functioning (RF2) 84.8 (SD = 20.8), emotional functioning (EF) 89.7 (SD = 13.0), cognitive functioning (CF) 89.9 (SD = 14.5), and social functioning (SF) 79.6 (SD = 25.9)—and on symptom scales—overall symptom score (SS) 15.5 (SD = 12.5), fatigue (FA) 25.6 (SD = 20.2), pain (PA) 14.4 (SD = 20.9), and nausea and vomiting (NV) 5.5 (SD = 10.9). Quality of life and global health score (GHS) was rated as 68.4 (SD = 23.6). Six separate questions assessed dyspnea (DY) 11.5 (SD = 20.1), insomnia (SL) 27.1 (SD = 27.9), appetite loss (AP) 7.5 (SD = 17.6), constipation (CO) 10.9 (SD = 24.3), diarrhea (DI) 19.0 (SD = 27.8), and financial difficulties (FI) 7.6 (SD = 17.5).

In the QOL-ANL 27 questionnaire the following total mean scores were achieved: in the symptom scales—bowel symptoms non stoma 27.5 (SD=19), bowel symptoms stoma 11.9 (17.2), stoma care 7.9 (SD = 11.4), pain 10.5 (SD = 13.9), and vaginal symptoms 28.9 (SD = 34.9). The single-item symptom scales assessed urinary frequency 26.4 (SD = 30.8), swelling in legs/ankles 8.0 (SD = 20.8), need to be close to a toilet 22.4 (SD = 27.3), cleaning oneself more often 25.3 (SD = 31.8), planning activities 17.8 (SD = 28.5), painful intercourse 12.3 (SD = 24.4), erectile problems 11.1 (SD = 24.8), and in two single-item functional scales evaluating sex life and interest—men (SEX M) 45.2 (SD = 23) and women (SEX F) 45.5 (SD = 19).

Radiotherapy technique (3D-CRT vs. IMRT), clinical stage (I/II vs. III), and time since radiotherapy did not affect quality of life statistically significantly (Supplementary materials—Table 2 and 3).

**Fig. 11:**
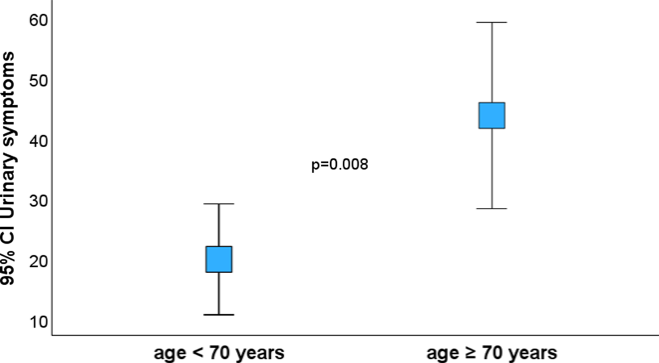
Urinary symptoms. *CI *confidence interval

**Fig. 12:**
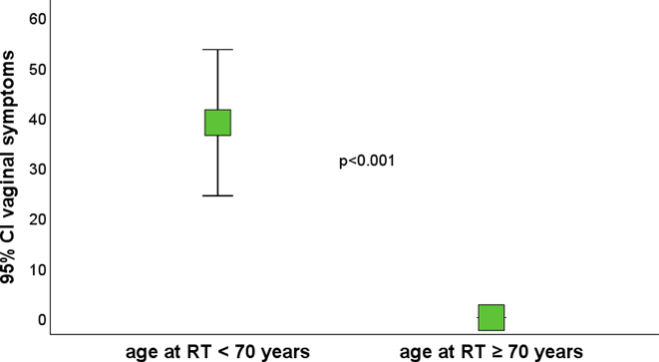
Vaginal symptoms. *CI *confidence interval, *RT* radiotherapy

A statistically significant negative influence of age higher than 70 was found on physical functioning (PF2; *p* = 0.022), global health score (GHS; *p* = 0.023), and urinary symptoms (*p* = 0.008; Fig. 11). Interestingly, intercourse symptoms (*p* = 0.001) and vaginal symptoms (*p* < 0.001) were better in the group of patients older than 70 (Fig. 12).

Age at questionnaire evaluation was found to be the most important factor affecting quality of life (both at the start of radiotherapy and at the questionnaire evaluation), and the explanation is discussed below.

## Discussion

The aim of our study was to evaluate and report real clinical data from patients treated with radio-/chemotherapy and ISBT boost for AC in terms of treatment outcomes, toxicity, and quality of life (QoL). Our findings show that patients in our cohort achieved high local tumor control, OS, and CFS. Age was found to be the most important factor affecting patents’ QoL.

Based on the Nigro protocol [15], randomized phase III trials established chemoradiotherapy as the standard approach in the treatment of localized and locally advanced AC [16–21]. A German meta-analysis of 41 trials [22] demonstrated that the combination of RT and chemotherapy with 5‑FU and MMC achieves the best results in terms of overall survival, disease-free survival, and CFS. No significant difference in clinical outcomes was seen in randomized controlled trials (RCT) where MMC was replaced by cisplatin except for DFS with less hematological toxicity. Induction therapy with cisplatin and 5‑FU is not recommended outside clinical trials. Surgery alone may be considered in stage I AC of the anal verge region, whereas RT alone with 5‑FU is considered in the older and frail population [30]. 5‑FU can be substituted by oral capecitabine without affecting treatment outcomes as demonstrated in several phase II trials [31–33]. Definitive radiochemotherapy (RT-CT) with 5‑FU and MMC provides locoregional control rates of 68–84%, 5‑year overall survival rates of 65–79%, and colostomy-free survival rates of 65–75% according to two of the major RCT [18, 19].

The optimal RT dose for curative RT-CT remains unclear. Based on clinical data, dose escalation and a radiation boost to the primary tumor or metastatic lymph nodes is currently the standard approach for locally advanced disease [26, 27, 34]. A tumor control probability model for AC suggests that dose reduction for early-stage AC could be beneficial [35]. The ongoing ACT4 phase II trial from PLATO compares a standard dose of RT with a deescalating approach. Enrolment has been completed and results are pending [36].

There is no randomized trial available to compare EBRT and a BT boost, and data on comparative effectiveness come from indirect estimates and retrospective studies only.

A prospective multicenter observational French study (FFCD-ANABASE) evaluated 1015 patients treated for AC between 2015 and 2020 [24]. 43% of patients had early-stage disease, 80% of patients were irradiated with IMRT, and 80% were referred for mitomycin-based concomitant RT-CT. In 15% of patients, interstitial brachytherapy (ISBT) was used to boost the primary tumor. With a median follow-up of 35.5 months, early-stage patients achieved DFS 84.3%, CFS 85.6%, and OS 91.7% at 3 years; advanced-stage patients achieved DFS 64.4%, CFS 66.0%, and OS 78.2% (*p* < 0.001). In multivariate analysis, male sex, locally advanced disease, and PS higher than 1 were associated with worse outcomes (DFS, CFS, OS). Use of the IMRT technique resulted in improved CFS in the whole group, boost using ISBT resulted in improved DFS in univariate analysis.

Frakuli published a systematic review of 10 retrospective studies from 1970 to 2014 with 1100 patients [37]. All studies were classified to provide a level of evidence graded as 3 according to the SIGN classification. 40–70% of patients in the study were in a low stage (T1.2 N0). Techniques, doses, and concomitant chemotherapy varied between studies, with the most common dose being 45 Gy in 25 fractions followed by an ISBT boost. The time between EBRT and ISBT was 27–56 days. Only one study used HDR ISBT with a dose per boost of 2 × 12 or 2 × 8 Gy. At 5 years, the following rates were achieved: LC/LRC, DFS, OS, and CFS: 79% (71–92%), 76% (66–86%), 69% (63–82%), and 76% (61–86%), respectively. A comparison between ISBT and EBRT was carried out in the systematic review by Gryc et al. Any differences in locoregional recurrences (24.0% vs. 19.0%), CFS (76.1% vs. 82.1%), OS (64% vs. 69%), and grade 4 late proctitis (2.0% vs. 1.0%) between ISBT and EBRT boost were reported [38].

Boost using HDR BT was examined in a meta-analysis of studies published by Canadian authors [29]. 10 studies with 448 patients treated between 1998 and 2019 were included. 371 patients (82.8%) were treated radically with EBRT and boost with ISBT or intracavitary BT, with a median dose of 6 Gy in 2 fractions; 77 patients (17.2%) in 2 studies [35, 39, 40] were indicated for boost with EBRT; and 345 patients (77%) were treated with concomitant RT-CT. Pooled analysis in this review suggests excellent clinical outcomes with limited toxicity. At 5 years, LC/LRC, DFS, OS, and CFS were 81%, 77.3% (SD 6.6%, R: 66–100%), 82.5% (SD 13.7%, R: 70–87.7%), and 80.4%, respectively.

Regarding toxicity, the most common acute forms during AC treatment include skin, genitourinary, gastrointestinal, and hematological. G3–4 toxicity was reported in 21% in RTOG 98–11 [18] and in 15.7% in the RTOG 0529 study [25], which already used modern RT techniques (IMRT). In a meta-analysis of HDR boost studies, only 9 patients developed this degree of toxicity in 2 of 10 studies [29]. In a direct comparison to a study published by Oehler-Janne et al. [39], toxicity with an ISBT boost was significantly lower than with an EBRT boost: 43% versus 15% (*p* = 0.008). However, late toxicity, mainly gastrointestinal, is a far more critical problem in the case of AC, including anal sphincter dysfunction, incontinence, proctitis, and treatment-related stoma, which significantly affect the QoL of cancer survivors. Bentzen et al. [41] evaluated fecal incontinence in 107 patients compared with 259 healthy volunteers: 43% of the patients developed a form of incontinence and 64% suffered from urgency. These difficulties correlated with lower global quality of life scores. Patients with more locally advanced tumors also had higher incontinence scores. Anal incontinence directly correlates with the dose to pelvic floor muscles including the internal and external sphincter [42]. Boosting with ISBT allows dose reduction to the area of the unaffected sphincter. In the study by Cagetti et al. [43], the doses to the normal sphincter were D_2cc_ 55.1 ± 10.7 Gy, D_90_ 45.4 ± 1.8 Gy, and D_mean_ 48.5 ± 2.8 Gy and resulted in fecal incontinence in 7.7% of patients at maximum G2. In the previously mentioned meta-analysis of HDR boost studies [29], G2 fecal incontinence occurred in 2.5–9% and proctitis in 2.5–19%. G3/4 toxicity was reported in 2.2–7.1% of patients, CFS in 80.4%, and the mean sphincter preservation rate was 88%.

In our cohort only 6 patients (6%) experienced G3 late gastrointestinal toxicity. Any type of toxicity was statistically significantly influenced by the EBRT technique (3DCRT vs. IMRT).

As a potentially long-term curable disease, late therapeutic toxicities such as chronic diarrhea, fecal incontinence, and urinary and sexual dysfunction, affect the long-term quality of life (QoL) of survivors [44–46]. Assessment of patients’ QoL is a very important part of the overall management of AC. The patient’s subjective view frequently does not correlate with the physician’s assessment of toxicity [47].

No validated questionnaire for objective assessment of QoL in AC was available until recently. Therefore, the European Organization for Research and Treatment of Cancer (EORTC) core questionnaire (QLQ-C30) [48] supplemented by a questionnaire for other cancers, e.g., colorectal cancer (QOL-CR29), was used in studies. Recently, a specific questionnaire for patients treated with RT-CT for AC has been validated—the EORTC QLQ-ANL27 [49], including a Czech version.

A meta-analysis of studies assessing the QoL of patients with AC was published by Sodergren et al. in 2015 [49]. In 11 studies, the EORTC QOL-C30 baseline questionnaire or the EORTC QOL-CR29 colorectal cancer assessment questionnaire was the most commonly used questionnaire for assessment. Bowel symptoms, diarrhea, and sexual problems had the greatest impact on QoL. The main objective of this study was to develop a questionnaire specific to this diagnosis that could be used in future studies.

The specific QOL-ANL-27 questionnaire, together with the baseline questionnaire, has already been used in the study by Sauter et al. [50]. The aim of the study was to assess QoL after RT-CT of AC and to find disease-, patient-, and treatment-related factors that influence QoL. Included in the study were 52 patients treated between 2004 and 2018 and QoL was assessed using the EORTC QLQ-C30 and QLQ-ANL27 questionnaires. The results were compared with the reference data from the German population. In the QLQ-C30 questionnaire, patients after AC therapy achieved significantly lower scores in role 70.5 vs. 82.7 (−12.2 points), emotional 70.5 vs. 77.1 (−6.6 points), and social functioning 83 vs. 89.8 (−6.8 points) but higher scores in cognitive functioning 95.8 vs. 88.6 (+7.2 points) as well as overall global health 77.6 vs. 63.4 (+14.2 points), diarrhea (+36.3 points), and constipation (+13.3 points) than the German population. Global QoL was affected by age over 70 and follow-up over 71 months, whereas there was no effect of disease or treatment. In the QLQ-ANL27, patients with relapsed disease had higher symptom scores on all the scales in comparison to patients in remission except for urinary frequency. Compared with 3D conformal RT, patients irradiated with the IMRT technique had higher scores in non-stoma bowel function (+23.3 points) and female sexual functioning (+24.2 points) and lower scores in the symptom scales of pain (−35.9 points), necessity of toilet proximity (−28.6 points), and cleanliness (−26.2 points). Global QoL was most affected by the role and physical functioning and fatigue scores.

Regarding the EORTC QLQ-C30, our study showed similar result as the study above in physical functioning scale (PF2) 84.8 vs. 82.3; higher scores in role functioning (RF2) 84.8 vs. 70.5 and emotional functioning (EF) 89.7 vs. 70.5; and lower scores in cognitive functioning (CF) 89.9 vs. 95.8 and social functioning (SF) 79.6 vs 83. There was also a lower overall GHS score of 68.4 vs. 80.2. In single-item symptom scales, our cohort showed better scores for constipation (10.9 vs. 18.6) and diarrhea (19 vs. 38.5). The scores in the QLQ-ANL27 questionnaire were comparable to the German study. Sexual functioning was significantly worse in both men and women in our cohort, men 45.2 (SD = 23) and women 45.5 (SD = 19), which may be caused to some extent by a more advanced age in our cohort, the median being 73 years (range 52–92) vs. 64.5 years (range 48–87) in the German study. Disease-, patient-, and treatment-related factors (stage I/II vs. III; RT technique 3DCRT vs. IMRT) did not have a statistically significant effect on most of the scores in either questionnaire. A statistically significant negative influence of age higher than 70 at the time of radiotherapy was found on physical functioning (PF2; *p* = 0.022), global health score (GHS; *p* = 0.023), and urinary symptoms (*p* = 0.008). Interestingly, intercourse symptoms (*p* = 0.001) and vaginal symptoms (*p* < 0.001) were better in the group of patients older than 70 (both at radiotherapy start and questionnaire evaluation), which could be explained by women maintaining sexual activity over 70 having an exceptional quality of life regarding these symptoms in comparison to younger women. This fact cannot be generalized because of the small number of patients answering the sexual part of the questionnaires and due to the fact that only individuals without advanced comorbidities who were able to undergo general anesthesia were selected for brachytherapy boost.

To our knowledge, our study is the first to evaluate QoL in AC patients treated with radiochemotherapy and a boost using interstitial HDR brachytherapy. Patient-reported outcomes should always be a part of a study because of the often-different toxicity assessments by physicians and patients. A limitation of the study is certainly the retrospective and only one-time QoL assessment without the possibility of comparison with pretreatment baseline scores. The long-term follow-up of the whole cohort is a positive feature.

## Conclusion

Boost using interstitial HDR brachytherapy is an established and safe part of anal cancer treatment. It allows important anatomical structures to be spared and leads to reduced gastrointestinal toxicity, which significantly affects the quality of life of patients with this diagnosis.

## Supplementary Information

Tables 2 and 3
