# Supplementary material for: Interstitial HDR brachytherapy for anal cancer—results and quality of life
Source: Strahlenther Onkol. 2024 Nov 14;201(7):687–98. doi: 10.1007/s00066-024-02316-5 (PMC12181215; doi:10.1007/s00066-024-02316-5)
Supplement: Supplementary file 1 — Tables 2 and 3 [file 66_2024_2316_MOESM1_ESM.docx]

Table 2 EORTC QOL – C30 v.3 Questionnaire

| Item | Total mean | CS | | RT technique^#^ | | Age at the time of completing the questionnaire | | Time since therapy | |
| --- | --- | --- | --- | --- | --- | --- | --- | --- | --- |
|  | N=58 | I, II  N=35 | III  N=23 | 3DCRT  N=17 | IMRT  N=40 | ˃70 y  N=31 | ≤ 70 y  N=27 | ˃5 y  N=36 | ≤ 5y  N=22 |
| FS | 86.2 (12.6) | 86,9(10,6) | 85(15,1) | 84,3(15,6) | 86,9(11,2) | 82,5(13,5)* | 90,7(10,2)* | 86,5(12,8) | 85,7(12,3) |
| PF2 | 84.8 (16.8) | 86,7(13,5) | 82(20,5) | 82,7(20,1) | 85,5(15,3) | 79,4(17,6)* | 91,5(13,6)* | 84,3(17,4) | 85,8(15,7) |
| RF2 | 84.8 (20.8) | 86,2(18,9) | 82,6(23,3) | 78,4(27,3) | 87,5(17) | 81,5(26,7) | 86,5(18,3) | 85,2(21) | 84,1(20,4) |
| EF | 89.7 (13.1) | 85,6(12,5) | 91,3(13,8) | 88,7(14,8) | 90,0(12,4) | 88,5(14,5) | 91,0(11,5) | 90(13,1) | 89(12,9) |
| CF | 89.9 (14.5) | 88,6(16,3) | 92,0(10,8) | 93,1(10) | 88,8(16) | 86,5(17,7)* | 94,2 (8,0) * | 90,7(12) | 88,6(17,7) |
| SF | 79.6 (25.9) | 82,9(20,9) | 74,6(31,4) | 75,5(35,3) | 81,3(20,8) | 72,9(29,9)* | 87,8(18,0)* | 81(26,7) | 77,3(24,4) |
| SS | 15.5 (12.5) | 15,6(11,2) | 15,4(14,1) | 17,7(13,5) | 14,7(12) | 18,3(11,1) | 12,0(13) | 15,2(11,5) | 16,1(13,9) |
| FA | 25.6 (20.2) | 26,7(19,2) | 24,2(21,6) | 30,7(22) | 23,6(19,3) | 31,2(18,8)* | 18,8(20,6)* | 25,6(19,4) | 25,8(21,6) |
| NV | 5.5 (10.9) | 5,2(9,6) | 5,8(12,6) | 9,8(12,9) | 3,8(9,5) | 6,3(11,0) | 4,5(11,1) | 6(10,5) | 4,6(11,4) |
| PA | 14.4(20.9) | 13,3(17,7) | 15,9(24,8) | 13,7(21,6) | 15 (20,7) | 16,7,2(22,0) | 11,5(19,9) | 13(18,5) | 16,7(24,1) |
| DY | 11.5 (20.1) | 9,5(18,8) | 14,5(21,6) | 13,7(20) | 10,8(23,7) | 14,6 (22,3) | 7,7(17,1) | 9,3(16,9) | 15,2(24) |
| SL | 27.1 (26.4) | 28,8(29,8) | 24,6(24,5) | 23,5(22,2) | 27,7(29,6) | 30,4(30,7) | 23,0(24,5) | 26,1(28,3) | 28,8(27,1) |
| AP | 7.5 (17.6) | 6,7(19,2) | 8,7(14,6) | 11,8(25,4) | 5,8 (12,7) | 9,4(21,1) | 5,1(12,2) | 8,3(19,8) | 6(12,9) |
| CO | 10.9 (24.3) | 13,3(27,8) | 7,25(16,9) | 5,9(17) | 13,3(26,7) | 11,5(27,6) | 10,2(21,6) | 8,3(25,3) | 15,2(21,9) |
| DI | 19.0 (27.8) | 19,1(30,1) | 18,8(23,7) | 21,6(34,2) | 18,3(24,7) | 22,9 (32,2) | 14,6(22,3) | 19,4(28,7) | 18,2(26) |
| FI | 7.6 (17.5) | 5,7(14,9) | 10,5(20,6) | 14,3(25,5) | 5,0(11,9) | 8,3(18,9) | 6,6(16,3) | 9,5(20,1) | 4,6(11,4) |
| GHS | 68.4 (23.6) | 66,7(21,5) | 71(21,9) | 59,8(30,5) | 72 (19,2) | 54,7(24,6) * | 82,4(14,6)* | 65,5(26,3) | 74(17) |

^#^ One patient treated with brachytherapy only

* Statistically significant

Standard deviation inside the parentheses.

Abbreviations: CS=clinical stage, RT=radiotherapy, 3DCRT = three-dimensional conformal radiotherapy, IMRT=intensity modulated radiotherapy, FS=overall functional score, PF2=physical functioning, RF2=role functioning, EF=emotional functioning, CF=cognitive functioning, SF=social functioning, SS=overall symptom score, FA=fatigue, NV=nausea and vomiting, PA=pain, DY=dyspnoea, SL= insomnia, AP= appetite loss, CO=constipation, DI= diarrhoea, FI=financial difficulties, GHS=global health score

Table 3 QOL-ANL 27 Questionnaire

| Item | Total mean (SD) | CS | | RT technique # | | Age at the time of completing the questionnaire | | Time since therapy | |
| --- | --- | --- | --- | --- | --- | --- | --- | --- | --- |
|  |  | I, II | III | 3DCRT | IMRT | ˃70 y | ≤ 70 y | ˃ 5 y | ≤ 5y |
| Bowel symptoms | 27,5 (19)  N=51 | 31,2 (21)  N=31 | 24,5 (18,2)  N=20 | 32,9 (20,9)  N=15 | 26,8 (19,6)  N=36 | 33(21.6)  N=32 | 21.0(14.9)  N=26 | 28,2 (19,3)  N=32 | 29, 1(21,5)  N=19 |
| Pain | 10,5(13,9)  N=58 | 11,1(15,7)  N=35 | 9,7(10,4)  N=23 | 9,2 (16,7)  N=17 | 11,4(12,5)  N=40 | 12.5 (16.7)  N=32 | 8.1 (9.6)  N=26 | 9,4 (14)  N=36 | 12,4 (13,3)  N=22 |
| Stoma care | 7,9 (11,4)  N=7 | NA | NA | NA | NA | NA | NA | NA | NA |
| SEX F | 45,5 (19)  N=39 | 68,9(27,5)  N=23 | 69,8(27,2)  N=16 | 56,6(22,8)  N=11 | 73,3(27)  N=27 | 78,7(19)  N=18 | 61,2(30,6)  N=18 | 66,8(27,5)  N=25 | 73,8(26,6)  N=14 |
| SEX M | 45,2(23)  N=7 | NA | NA | NA | NA | NA | NA | NA | NA |
| Urinary frequency | 26,4 (30,8)  N=58 | 30,5(31,2)  N=35 | 20,3(29)  N=23 | 33,3(37,9)  N=17 | 22,5(26,2)  N=40 | 39.6(33.3) *  N=32 | 10.3(18.3) *  N=26 | 29,6(33,1)  N=36 | 21,2(25,7)  N=22 |
| Swelling in legs/ankles | 8(20,8)  N=58 | 4,8(14,1)  N=35 | 13,0(27,3)  N=23 | 13,7(28,14)  N=17 | 5,8(16,5)  N=40 | 9.4(22.8)  N=32 | 6.4(18.9)  N=26 | 10,2(23,3)  N=36 | 4,6(15,2)  N=22 |
| Need to be close to a toilet | 22,4(27,3)  N=58 | 23,8(30,4)  N=35 | 20,3(21,4)  N=23 | 27,5(34,7)  N=17 | 20(23,3)  N=40 | 30.2(32) *  N=32 | 14.1 (16.8) *  N=26 | 24(30)  N=36 | 19,7(21,7)  N=22 |
| Clean more often | 25,3 (31,8)  N=58 | 29,5(33,6)  N=35 | 18,8(27,5)  N=23 | 25,5(38,8)  N=17 | 25(28,6)  N=40 | 33.3(34.9) *  N=32 | 15.4(25.4) *  N=26 | 25(33,7)  N=36 | 25,8(28,3)  N=22 |
| Planning activities | 17,8(28,5)  N=58 | 18,0(30,2)  N=35 | 17,4(25,8)  N=23 | 29,4(37,7)  N=17 | 13,3(22,1)  N=40 | 28.1(34.0) *  N=32 | 7.7(17.1) *  N=26 | 20,4(30,7)  N=36 | 13,6(23,9)  N=22 |
| Painful intercourse | 12,3(24,4)  N=46 | 10,7(23,7)  N=28 | 14,8(25,4)  N=18 | 18(24,9)  N=13 | 10,4(24,2)  N=32 | 4.8(15.9) *  N=21 | 21.2(30.0) *  N=22 | 11,5(21,9)  N=29 | 13,7(28,1)  N=17 |
| Vaginal symptoms. | 28,9(34,9)  N=35 | 28(35,7)  N=21 | 30(33,4)  N=14 | 44,5(37,6)  N=11 | 21,8(31)  N=24 | 11.8(26.5) *  N=17 | 45(35.6) *  N=18 | 32,8(35,5)  N=22 | 22,2(32,6)  N=13 |
| Erectile problems | 11,1(24,8)  N=6 | NA | NA | NA | NA | NA | NA | NA | NA |

^#^ One patient treated with brachytherapy only

^*^ Statistically significant

NA Not applicable

Standard deviation inside the parentheses.

Abbreviations: SD=standard deviation, CS=clinical stage, RT=radiotherapy, 3DCRT = three-dimensional conformal radiotherapy, IMRT=intensity modulated radiotherapy
